# IL-23-mediated mononuclear phagocyte crosstalk protects mice from *Citrobacter rodentium*-induced colon immunopathology

**DOI:** 10.1038/ncomms7525

**Published:** 2015-03-12

**Authors:** Tegest Aychek, Alexander Mildner, Simon Yona, Ki-Wook Kim, Nardy Lampl, Shlomit Reich-Zeliger, Louis Boon, Nir Yogev, Ari Waisman, Daniel J. Cua, Steffen Jung

**Affiliations:** 1 Department of Immunology, Weizmann Institute of Science, Rehovot 76100, Israel; 2 Bioceros, Yalelaan 46, 3584 CM Utrecht, The Netherlands; 3 Institute for Molecular Medicine, University Medical Center of the Johannes Gutenberg University, 55131 Mainz, Germany; 4 Merck Research Laboratories, 901 South California Avenue, Palo Alto, California 94304-1104, USA

## Abstract

Gut homeostasis and mucosal immune defense rely on the differential contributions of dendritic cells (DC) and macrophages. Here we show that colonic CX_3_CR1^+^ mononuclear phagocytes are critical inducers of the innate response to *Citrobacter rodentium* infection. Specifically, the absence of IL-23 expression in macrophages or CD11b^+^ DC results in the impairment of IL-22 production and in acute lethality. Highlighting immunopathology as a death cause, infected animals are rescued by the neutralization of IL-12 or IFNγ. Moreover, mice are also protected when the CD103^+^ CD11b^−^ DC compartment is rendered deficient for IL-12 production. We show that IL-12 production by colonic CD103^+^ CD11b^−^ DC is repressed by IL-23. Collectively, in addition to its role in inducing IL-22 production, macrophage-derived or CD103^−^ CD11b^+^ DC-derived IL-23 is required to negatively control the otherwise deleterious production of IL-12 by CD103^+^ CD11b^−^ DC. Impairment of this critical mononuclear phagocyte crosstalk results in the generation of IFNγ-producing former TH17 cells and fatal immunopathology.

## Abstract (web summary)

Macrophages and dendritic cells contribute to gut homeostasis and mucosal immune defense. Here, Aychek *et al*. describe an IL-23-based crosstalk between these cells that prevents lethal immunopathology during *Citrobacter rodentium* infection.

## Graphical abstract

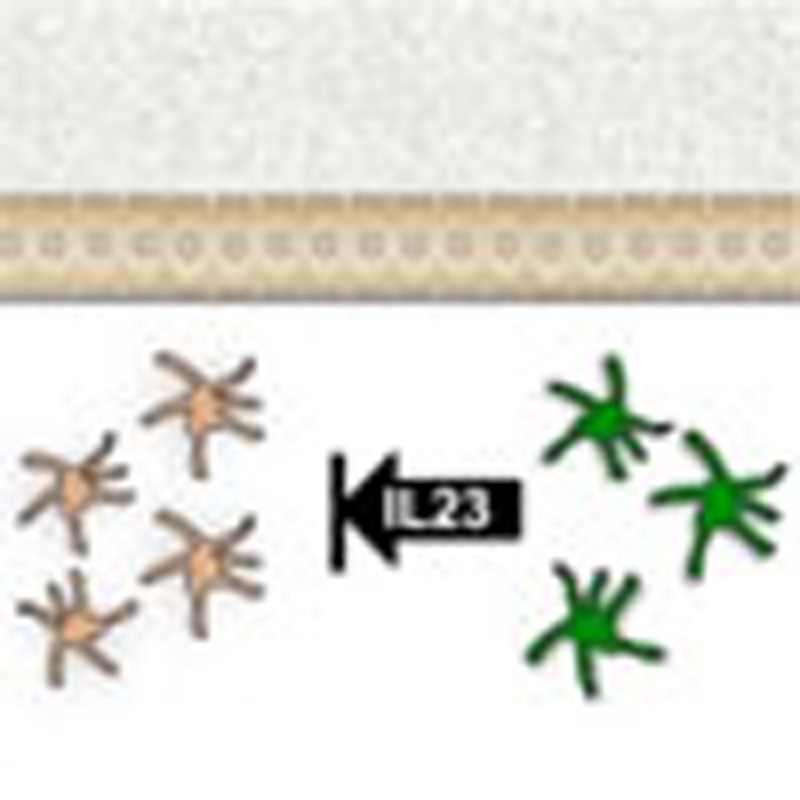

The intestinal lamina propria hosts a complex make-up of mononuclear phagocytes, including dendritic cells (DC) and macrophages that collectively contribute to the maintenance of gut homeostasis and responses to pathogen challenges^1^. Murine intestinal DC subpopulations are currently defined by the expression of integrins CD11c (α_X_), CD11b (α_M_) and CD103 (α_E_β7), as well as CD24 (ref. 2). CD103^+^ CD11b^−^ DC depend, like classical splenic XCR1^+^ CD8α^+^ DC, on the transcription factors *Irf8*, *Id2*, and *Batf3* for their developmenton^3^. These cells migrate to the mesenteric lymph nodes to prime and polarize naïve T cells and seem as their splenic counterpart specialized in cross-presentation^4^. In contrast, CD103^+^ CD11b^+^ DC in the ileum were shown to be required for the efficient generation of Th17 cells^5^,^6^,^7^,^8^. In the colon, CD103^+^ CD11b^+^ DC are rare, but might be functionally replaced by a population lacking CD103 expression^9^. The ontogeny of CD103^+^ and CD103^−^ CD11b^+^ DC remains less well defined, as these cells are currently difficult to discern from monocyte-derived cells^10^. The most abundant intestinal mononuclear phagocytes are CX_3_CR1^hi^ macrophages that develop from Ly6C^+^ blood monocytes and are conditioned in the healthy gut to become non-inflammatory cells^11^,^12^. In steady state, these cells are non-migratory and are critically involved in the maintenance of gut homeostasis^13^. Specific activities of intestinal macrophages and the DC subsets, and in particular, potential crosstalk between these cells remain incompletely understood.

Attaching and effacing (A/E) enteropathogens adhere to intestinal epithelial cells of the infected host, efface their microvilli and reorganize their cytoskeleton. They control host cells by the injection of effector molecules via a type III secretion system, whose components are encoded on a conserved pathogenicity island^14^. In immunocompetent mice, the A/E pathogen *Citrobacter rodentium*, which serves as model organism for human enteropathogenic *Escherichia coli* and enterohaemorrhagic *E. coli*^15^, induces a transient, distal colitis followed by a systemic CD4^+^ T-cell-dependent IgG response resulting in bacterial clearance^16^. Mice deficient in the cytokine interleukin-23 (IL-23), a heterodimer of p19 and p40 polypeptide chains encoded by the *il23a* and *il12b* genes, respectively^17^, fail to control *C. rodentium* and die from the infection within a week^18^. IL-23 requirement has been linked to IL-22 secretion by RORγt-dependent innate lymphoid cell (ILC; now named group 3 ILC, or ILC3; ref. 19). Thus, *C. rodentium*-challenged *il23a*-deficient animals can be rescued by exogenous IL-22; moreover, also IL-22-deficient mice die from *C. rodentium* infection, albeit with a delay^20^. IL-22 is believed to boost epithelial regeneration and induces epithelial cells to express antimicrobial peptides (AMPs), including defensins and lectins of the RegIII family^20^,^21^.

Here we investigated the cellular source of early IL-23 that protects *Citrobacter*-challenged mice. In line with recent reports, we establish that IL-23 production by colonic CX_3_CR1^+^ mononuclear phagocytes, including CD103^−^ CD11b^+^ DC and macrophages—but not CD103^+^ CD11b^−^ DC—was required for the induction of IL-22 and AMPs. Animals harbouring an IL-23 deficiency acutely died from the *Citrobacter* challenge. Interestingly though, mice could be rescued by an additional IL-12 deficiency in the CD103^+^ CD11b^−^ DC compartment, as well as antibody-mediated neutralization of IL-12 and interferon-γ (IFNγ). Collectively, we establish that IL-23, in addition to its role as inducer of IL-22 and AMPs, suppresses IL-12 production by colonic CD103^+^ CD11b^−^ DC. Impairment of this mononuclear phagocyte crosstalk results in lethal immunopathology that is associated with the generation of IFNγ-producing TH1 cells from former TH17 cells.

## Results

### Haematopoietic IL-23 is essential to survive *C. rodentium* infections

**Figure 1:**
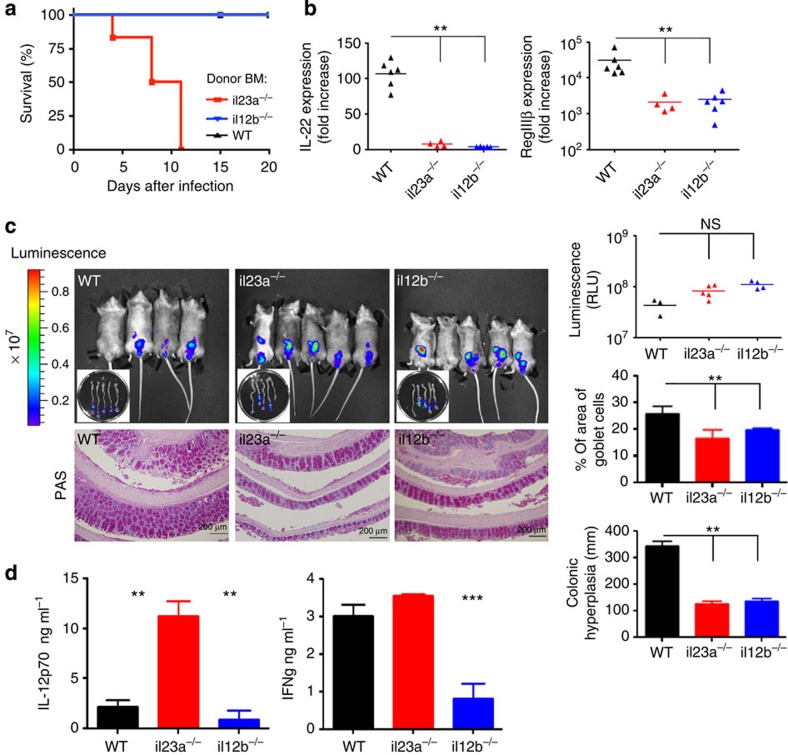
Haematopoetic IL-23 is essential to survive *C. rodentium* infections. (**a**) Survival curves of *C. rodentium*-challenged (*il12b*^−/−^>WT), (*il23a*^−/−^> WT) and (WT>WT) BM chimeras (*n*=6 per group, representative of three individual experiments). (**b**) Real-time PCR analysis of IL-22 and RegIIIβ expression in colonic tissues collected on day 7 PI. All groups, as in **a** (*n*=4–6 mice per group, representative of three individual experiments). Data were analysed by analysis of variance (ANOVA) followed by Tukey’s *post hoc* test, **P<0.01. (**c**) Tissue distribution of the luminescent *C. rodentium* strain at day 7. IVIS results are displayed as pseudo-colour images of peak bioluminescence, with variations in colour representing light intensity at a given location; right panel quantification of the luminescent *C. rodentium* signal. Lower panel, periodic acid-Schiff staining indicating goblet cell deletion, and decreased mucosal hyperplasia in (*il12b*^−/−^>WT) and (*il23a*^−/−^>WT) chimeras. (*n*=3–5 mice per group from three individual experiments). Data were analysed by ANOVA followed by Tukey’s *post hoc* test, **P<0.01. (**d**) ELISA for IL-12 and INFγ performed on supernatants of colon explant cultures of *C. rodentium*-challenged (*il12b*^−/−^>WT), (*il23a*^−/−^>WT) and (WT>WT) BM chimeras on day 7 PI (*n*=3–5 mice per group from three individual experiments). Data were analysed by ANOVA followed by Tukey’s *post hoc* test, **P<0.01, ***P<0.001.

Host defense against *C. rodentium* involves the orchestrated expression of cytokines and AMPs^20^. Kinetics of the response can, however, considerably vary depending on the local microbiota composition^22^. We therefore analysed the colonic tissue isolated from *Citrobacter*-challenged C57BL/6 (wild-type) WT mice housed in our animal facility for the expression of IL-12 superfamily cytokines, for example, IL-12 and IL-23, composed of p35/p40 and p19/p40, respectively. We found the expression of *il23a* (encoding IL23p19) to be transiently upregulated on day 3 post infection (PI), alongside *il12b* (encoding IL12p40), while *il12a* (encoding IL12p35) was induced only by day 6 PI (Supplementary Fig. 1a, Supplementary Table 1). As previously reported^20^, the induction of IL-23 was followed by the production of IL-22 and the AMP RegIIIβ, as well as members of the S100A family and IL-17A (Supplementary Fig. 1b,c). To probe the requirement of a haematopoietic IL-23 source, we generated chimeras by engrafting lethally irradiated WT mice with either *il12b*- or *il23a*-deficient bone marrow (BM) cells. (*il23a*^−/−^>WT) chimeras died from the *C. rodentium* challenge by day 12 PI; surprisingly though, (*il12b*^−/−^>WT) BM chimeras mice survived (Fig. 1a). Both the chimeras displayed an impaired induction of IL-22 and RegIIIβ transcripts, compared with controls (Fig. 1b). The absence of these factors alone could hence not explain the differential lethality. To determine whether BM chimeras harbouring the *il23a* mutation succumb to the infection due to increased pathogen burden, we took advantage of the bioluminescence of the *C. rodentium* strain we used for the challenge^23^. The reporter signal intensities emitted from chimeras lacking IL-23 or IL-12/23 production were comparable (Fig. 1c), arguing against bacterial overload as the death cause. Challenged (*il23a*^−/−^>WT) and (*il12b*^−/−^>WT) chimeras displayed reduced mucosal hyperplasia and goblet cell prevalence, as compared with (WT>WT) controls. However, these alterations were comparable in the two mutant groups (Fig. 1c). To gain further insight into the potential mechanism underlying the differential lethality of the chimeras, we measured cytokine levels in the supernatant of colon explant cultures by enzyme-linked immunosorbent assay (ELISA). Interestingly, samples of infected (*il23a*^−/−^>WT) but not (*WT*>WT) chimeras displayed a significant increase in IL-12, which was as expected absent from the (*il12b*^−/−^>WT) chimeras (Fig. 1d). On the other hand, IFNγ levels were increased in both (*il23a*^−/−^>WT) and (*WT*>WT) cultures, but low in (*il12b*^−/−^>WT) cultures. Thus collectively, increased IL-12 production in the colon correlates with lethality, as does IFNγ production, in absence of IL-22.

Of note, the fact that (*il12b*^−/−^>WT) chimeras survived the *C. rodentium* challenge contrasts the reported lethality observed for *il12b*^−/−^ mice^18^. Indeed, also in our animal facility these mice died from infection, as did (*il12b*^−/−^>*il12b*^−/−^) BM chimeras, while animals with p40-proficient donor or recipient genotype survived (Supplementary Fig. 2). This discrepancy might be related to a radio-resistant long-lived p40-expressing population or could result from the inherent difference between mice that lack p40 throughout development versus chimeras rendered p40 deficient only in adulthood. While this finding warrants further exploration, it does not affect our study as all subsequent experiments were performed in BM chimeras and, wherever possible, included internal controls.

Collectively, our data confirm the earlier notion that haematopoietic cells are a critical source of IL-23. Surprisingly though, the resistance of (*il12b*^−/−^>WT) BM chimeras to the *C. rodentium* challenge indicated that the IL-22 or AMP deficiency alone are not responsible for acute death. Rather our data suggest that the lethality might be due to IL-12 overproduction.

### CX_3_CR1^+^ cell-derived IL-23 is critical to survive the *C. rodentium* challenge

To further define the cell type that produces IL-23 in the infected colon, we employed a conditional cell ablation strategy targeting intestinal mononuclear phagocytes. CD11c-DTR mice^24^ allow for diphtheria toxin (DTx)-induced conditional ablation of the three major colonic mononuclear phagocytes, including CD103^+^ CD11b^−^ DC, CD103^−^ CD11b^+^ DC and CX_3_CR1^hi^ macrophages^25^.

**Figure 2:**
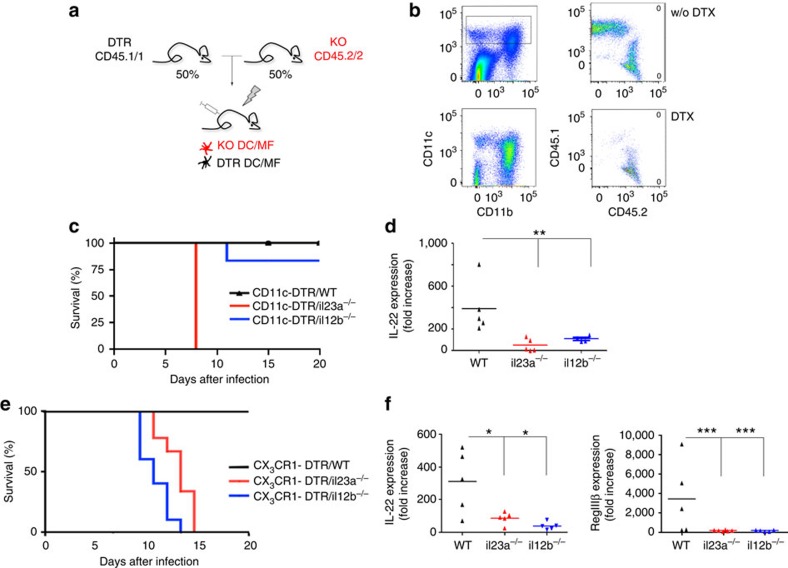
IL-23 production by colonic macrophages and CD103^−^ CD11b^+^ DC is required to survive the *C. rodentium* challenge. (**a**) Schematic of mixed BM chimera approach. (**b**) Flow cytometric analysis of colon of DTx-injected mouse on day 5 after DTX treatment (lower panel) and control (upper panel) showing the depletion of CD11c^+^ CD45.1^+^ DTR-transgenic cells. Data represent one of the three independent experiments (*n*=3 mice per group). (**c**) Survival curves of DTx-treated (*il23a*^−/−^ /CD11c-DTR>WT), (*il12b*^−/−^ /CD11c-DTR>WT) and (WT/CD11c-DTR>WT) chimeras following *Citrobacter* challenge (*n*=6 per group from three individual experiments). (**d**) Real-time PCR analysis of IL-22 expression in colonic tissues collected from DTx-treated (*il23a*^−/−^ /CD11c-DTR>WT), (*il12b*^−/−^ /CD11c-DTR>WT) and (WT/CD11c-DTR>WT) chimeras on day 7 PI (*n*=4–6 per group from three individual experiments). Data were analysed by analysis of variance (ANOVA) followed by Tukey’s *post hoc* test, **P<0.01. (**e**) Survival curves of DTx-treated (*il23a*^−/−^ /CX_3_CR1-DTR>WT), (*il12b*^−/−^/ CX_3_CR1-DTR>WT) and (WT/CX_3_CR1-DTR>WT) chimeras following *Citrobacter* challenge (*n*=7 per group from three individual experiments). (**f**) Real-time PCR analysis for IL-22 and RegIIIβ expression of colonic tissues collected from DTx-treated (*il23a*^−/−^/ CX_3_CR1-DTR>WT), (*il12b*^−/−^/ CX_3_CR1-DTR>WT) and (WT/CX_3_CR1-DTR>WT) chimeras on day 8 PI (*n*=5 per group from three individual experiments). Data were analysed by ANOVA followed by Tukey’s *post hoc* test *P<0.05, ***P<0.001. w/o, without.

To investigate the impact of IL-23 production by DC or macrophages on the anti-*Citrobacter* response, we took advantage of a mixed BM chimera approach^26^ (Fig. 2a). Specifically, we generated three groups of chimeras by reconstituting WT mice with equal mixtures of DTR-transgenic BM and mutant BM. Upon DTx treatment, such mixed chimeras are left with mutant mononuclear phagocytes only, while other cellular compartments remain composed of WT and mutant cells. Flow cytometric analysis of DTx-treated mixed BM chimeras confirmed the specific ablation of DTR-transgenic (CD45.1^+^) cells from the colon (Fig. 2b). DTx-treated (*il23a*^−/−^ /CD11c-DTR>WT) chimeras, retaining only IL-23-deficient CD11c^+^ cells, including all intestinal DC and macrophages, died by day 8 PI (Fig. 2c). This establishes DC or macrophages as critical IL-23 sources in the colon. Interestingly, infected DTx-treated (*il12b*^−/−^ /CD11c-DTR>WT) chimeras survived and the combined absence of IL-23 and IL-12 thus again conferred resistance to the challenge. Mice lacking either IL-23- or IL-12/23-producing CD11c^+^ cells displayed a significant reduction in IL-22 transcripts in colonic tissue, when compared with WT chimeras (Fig. 2d). None of the BM chimeras exhibited a significantly increased bacterial burden (Supplementary Fig. 3a).

Taking advantage of CX_3_CR1^*Cre*^ mice^27^, we next developed an ablation strategy that spares CD103^+^CD11b^−^ DC. Flow cytometric analysis of the ileum and colon of CX_3_CR1^*Cre*^:R26-RFP reporter mice revealed that CD64^+^ CD11b^+^ macrophages were, as expected, homogeneously labelled (Supplementary Fig. 4a,b; Supplementary Fig. 5a,b); also colonic CD103^−^ CD11b^+^ DC showed frequent rearrangements. Of note, ileal CD103^+^ CD11b^+^ DC were found to be efficiently labelled in CX_3_CR1^*Cre*^:R26-RFP reporter mice as well (Supplementary Fig. 5a,b); however, these cells are rare in the colon (Supplementary Fig 4a,b). In contrast, few ileal and colonic CD103^+^ CD11b^−^ DC showed signs of Cre recombinase activity. After crossing CX_3_CR1^*Cre*^ mice to iDTR animals^28^, CX_3_CR1-expressing cells become sensitive to DTx. CX_3_CR1^Cre^:iDTR (‘CX_3_CR1-DTR’) mice did not tolerate prolonged ablation, but (CX_3_CR1-DTR>WT) BM chimeras showed no adverse side effects of repetitive and prolonged DTx treatment. Flow cytometric analysis of both the colon and the ileum of DTx-injected CX_3_CR1-DTR chimeras revealed that—consistent with the above reporter gene expression profile—intestinal macrophages were efficiently ablated (Supplementary Fig. 4c–e; Supplementary Fig. 5c–e). In addition, colonic CD103^−^ CD11b^+^ cells and ileal CD103^+^ CD11b^+^ compartments were significantly affected. In contrast, intestinal CD103^+^ CD11b^−^ DC were largely resistant to the ablation. In summary, the CX_3_CR1Cre system spares (BatF3-dependent) CD103^+^ CD11b^−^ DC, but targets both CX_3_CR1^hi^ macrophages and CD103^+^ and CD103^−^ CD11b^+^ DC; the latter might be due to their expression of the chemokine receptor or their derivation from CX_3_CR1-expressing cells^27^. For simplicity we will refer from here on to cells that are in CX_3_CR1-DTR mice sensitive to DTx as ‘CX_3_CR1^+^ cells’. In analogy to the above CD11c-DTR experiment, we generated mixed chimeras with CX_3_CR1-DTR BM and either mutant or WT BM, and challenged the animals with *C. rodentium*. Mice that after DTx treatment specifically lacked *il23a* in their CX_3_CR1^+^ cell compartment died by day 10–12 after infection (Fig. 2e). Furthermore, also mice that lacked *il12b* in CX_3_CR1^+^ cells, impairing both their IL-12 and IL-23 production, died. Both the groups of animals displayed a considerable reduction in IL-22 and RegIIIβ transcripts, when compared with WT chimeras (Fig. 2f). These data establish that the production of the colonic IL-23, which induces IL-22 and AMPs in response to the *C. rodentium* challenge is restricted to CD103^−^ CD11b^+^ DC or macrophages, but not CD103^+^ CD11b^−^ DC. Our data thus support previous reports of IL-23 expression by these cells or their equivalents^8^,^29^,^30^,^31^. Importantly, the fact that DTx-treated (*il12b*^−/−^/CX_3_CR1-DTR>WT) chimeras die from the *Citrobacter* challenge, but (*il12b*^−/−^ /CD11c-DTR>WT) chimeras survive, indicates that the rescue results from the introduction of the IL-12 deficiency into the CD103^+^ CD11b^−^ DC compartment. These data support our earlier observation that exacerbated IL-12 production by CD103^+^ CD11b^−^ DC is associated with the lethality in our system (Fig. 1d).

### IL-23 regulates IL-12 production by CD103^+^ CD11b^−^ DC

**Figure 3:**
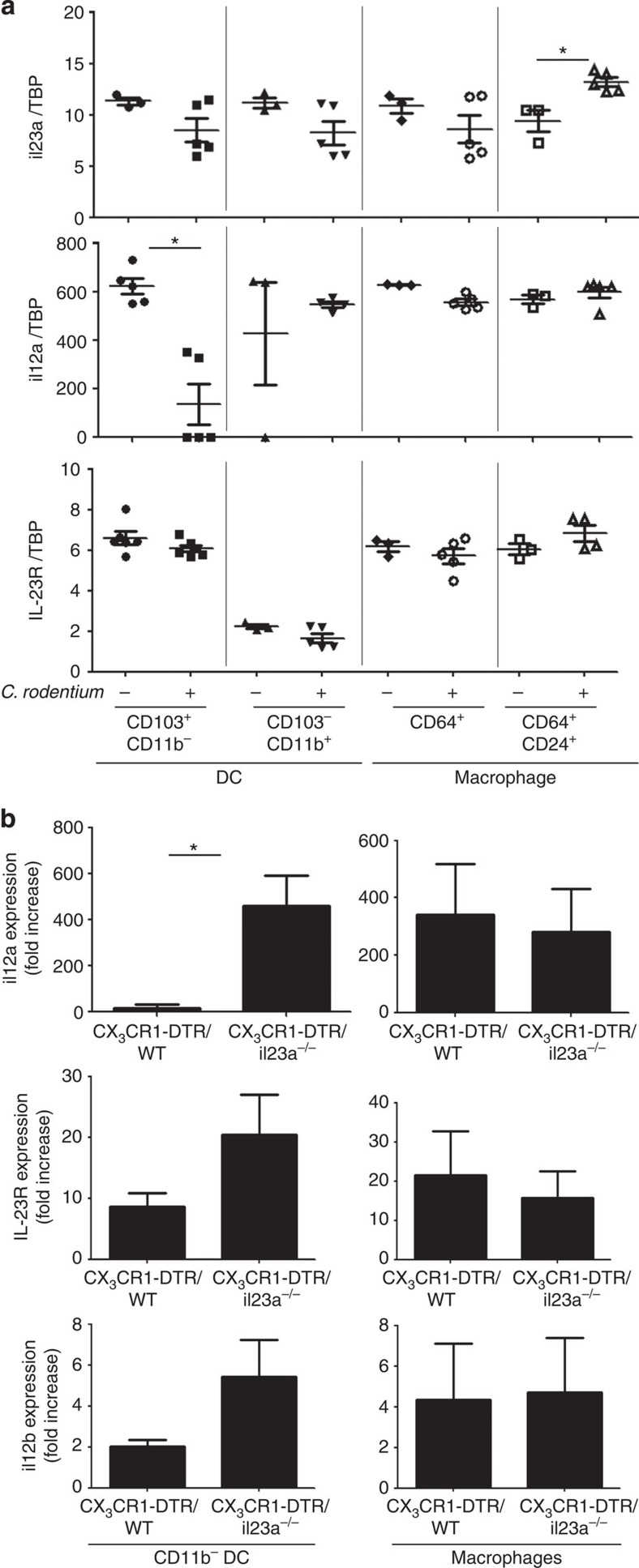
Analysis of intestinal mononuclear phagocytes for basal and PI cytokine production. (**a**) Reverse transcription PCR (RT–PCR) analysis of FACS-sorted lamina propria DC and macrophages isolated from uninfected or infected WT mice 3 days PI. Data are representative of 3–6 independent experiments with DC and macrophages pooled from 7–10 mice (for sorting strategy see Supplementary Fig. 6). (**b**) RT–PCR analysis of CD103^+^ CD11b^−^ CD11c^+^ DC and CD103^−^ CD11b^+^ CD11c^+^ cells, including macrophages and CD103^−^ CD11b^+^ DC were sorted from DTx-treated mixed (WT/CX_3_CR1-DTR>WT) and (*il23a*^−/−^/CX_3_CR1-DTR>WT) BM chimeras. Data are presented as fold increase compared with the DC from uninfected mice. Data were obtained from three independent experiments with cells pooled from 9–11 chimeras. Data were analysed by two-tailed *t*-test *P<0.05. TBP, TATA binding protein.

To directly define the cells that express IL-12 and IL-23 before and after *C. rodentium* challenge *in vivo*, we performed an reverse transcription PCR expression analysis on fluorescence-activated cell sorting (FACS)-sorted cells (Supplementary Fig. 6a,b). Colonic CD11c^+^ CD64^+^ CD24^+^ macrophages, but neither CD103^+^ CD11b^−^ DC nor CD103^−^ CD11b^+^ DC responded to the infection with *il23a* induction (Fig. 3a). CD103^+^ CD11b^−^ DC, on the other hand, downregulated *il12a* mRNA expression. Interestingly, CD103^+^ CD11b^−^ DC, but not CD103^−^ CD11b^+^ DC seem to express the IL-23 receptor, as they displayed signals for both *il23r* and *il12rb1* (Fig. 3a, Supplementary Fig. 6c) thus sensitizing these cells to IL-23. To examine whether the presence or absence of IL-23 expression from CX_3_CR1^+^ cells affects IL-12 production by CD103^+^ CD11b^−^ DC, we sorted these cells from infected DTx-treated (*il23a*^−/−^ /CX_3_CR1-DTR >WT) chimeras and compared them with cells isolated from infected DTx-treated control chimeras. In the absence of CX_3_CR1^+^ cell-derived IL-23, CD103^+^ CD11b^−^ DC displayed significantly enhanced *il12a* and *il12b* mRNA expression, whereas *il12a* mRNA expression by macrophages was unaffected (Fig. 3b).

**Figure 4:**
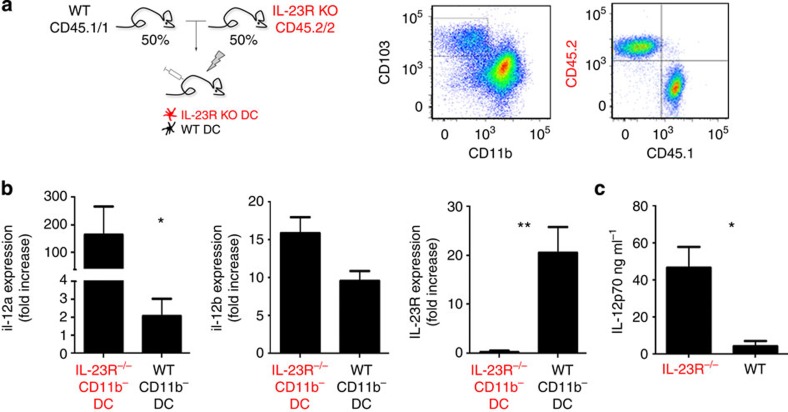
IL-23 negatively controls IL-12 production by colonic CD103^+^ CD11b^−^ DC *in vivo*. (**a**) Schematic of mixed chimera approach and flow cytometric analysis of colonic mononuclear phagocyte compartment of *Citrobacter*-infected (*il-23r*^*gfp/gfp*^ (CD45.2)/WT (CD45.1)>WT (CD45.2)) mice indicating chimerism of CD103^−^ CD11b^+^ DC population. Cells are gated according to scatter, doublets and CD11c expression. Data represent. one of the three independent experiments (*n*=3 mice per group). Data were analysed by two-tailed *t*-test *P<0.05. (**b**) reverse transcription PCR analysis of *il12a*, *il12b* and *il23R* transcripts measured in CD103^+^ CD11b^−^ CD11c^+^ DC sorted from (IL-23R^gfp/gfp^(CD45.2)/WT (CD45.1)>WT (CD45.2)) chimeras. (*n*=3 mice per group from three individual experiments). (**c**) ELISA for IL-12 performed on colon explant culture supernatants of*C. rodentium*-challenged (IL-23R^gfp/gfp^>WT) and (WT>WT) chimeras on day 7 PI (*n*=3–4 mice per group from three individual experiments). Data were analysed by two-tailed *t*-test *P<0.05, **P<0.01.

These data suggest that CD103^+^CD11b^−^ DC are subject to IL-23-dependent paracrine negative control. Specifically, colonic CX_3_CR1^+^ cells, including macrophages and possibly CD103^−^ CD11b^+^ DC produce IL-23 that suppresses IL-12 expression by CD103^+^ CD11b^−^ DC. To directly investigate this putative negative control circuit, we generated mixed chimeras with WT and *il-23r*^*gfp/gfp*^ BM^32^. The resulting mice harbour in their lamina propria both IL23R-deficient (CD45.2^+^) and -proficient (CD45.1^+^) CD103^+^ CD11b^−^ DC (Fig. 4a). We then challenged these mice with *C. rodentium*. Confirming the genotype of the BM grafts IL23R expression was absent from CD45.2^+^ CD103^+^ CD11b^−^ DC as compared with the CD45.1^+^ WT DC equivalents (Fig. 4b). More importantly though and proving that CD103^+^CD11b^−^ DC are in the infected mice under negative control by IL-23, IL23R-deficient DC expressed significantly more *il12a* mRNA than WT DC isolated from the same animals (Fig. 4b). In further support of the critical role of IL-23 in controlling IL-12 production, supernatants of cultured colon explants of *Citrobacter*-infected (*il23r*^−gfp/gfp^>WT) mice showed significantly higher IL-12 levels than that of infected controls (Fig. 4c). Collectively, this establishes that intestinal CD103^+^CD11b^−^ DC are target of a critical IL-23-mediated negative control circuit that prevents overt and potentially deleterious IL-12 production by these cells.

### IL-23 is required to prevent IFNγ-driven immunopathology

**Figure 5:**
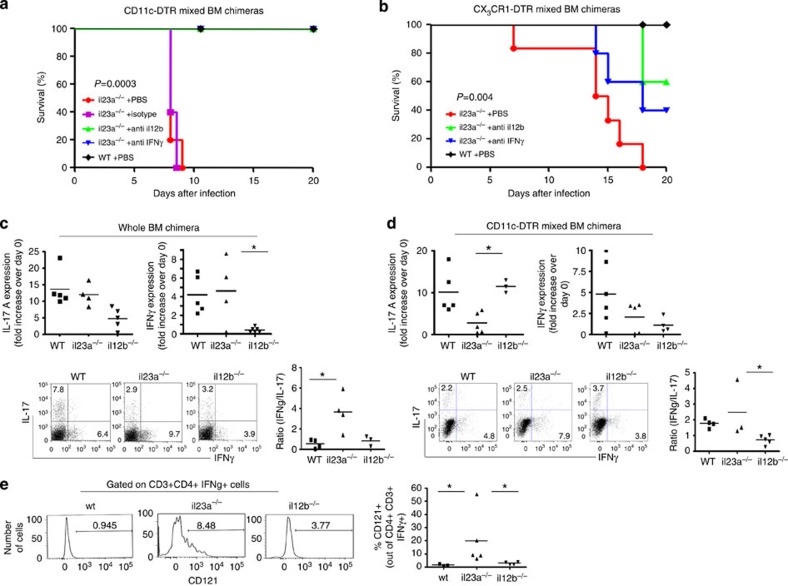
*Citrobacter*-infected mice that lack IL-23-producing macrophages and CD103^−^ CD11b^+^ DC succumb to IL-12- and IFNγ-mediated immunopathology. (**a**) Survival curves of DTx-treated mixed (*il23a*^−/−^/CD11c-DTR>WT) BM chimeras infected by *Citrobacter* gavage and treated with IFNγ- and IL12p40-specific neutralizing antibodies by intravenous injection or PBS and isotype control antibodies. (*n*=5 per group, representative of three individual experiments). Statistical analysis was performed using the log**-rank test. (**b**) Survival curves of DTx-treated mixed (*il23a*^−/−^/CX_3_CR1-DTR>WT) BM chimeras infected by *Citrobacter* gavage and treated with IFNγ- and IL12p40-specific neutralizing antibodies by intravenous injection or PBS; (*n*=5–6 per group from three individual experiments). Statistical analysis was performed using the log**-rank test. (**c**) (Top) real-time PCR analysis of IFNγ and IL-17A of colonic tissues collected on day 7 PI and (bottom) flow cytometric analysis of TH17 and TH1 isolated from the lamina propria of *il23a*^−/−^, *il12b*^−/−^ and WT BM chimeras. Cells were pregated on CD3^+^CD4^+^ cells. (*n*=3–6 mice per group from two individual experiments). Data were analysed by analysis of variance (ANOVA) followed by Tukey’s *post hoc* test *P<0.05. (**d**) (Top) real-time PCR analysis of IFNγ and IL-17A of colonic tissues collected on day 7 PI and (bottom). Flow cytometric analysis of TH17 and TH1 isolated from the lamina propria of indicated chimeras generated with haematopoietic mixture of mutant BM with CD11c-DTR BM. Cells were pregated on CD3^+^ CD4^+^ cells. (*n*=4–5 mice per group from two individual experiments). Data were analysed by ANOVA followed by Tukey’s *post hoc* test *P<0.05. (**e**) Flow cytometric analysis of TH1 cells isolated from DTx-treated (*il23a*^−/−^/CD11c-DTR>WT), (*il12b*^−/−^/CD11c-DTR>WT) and (*WT*/CD11c-DTR>WT) BM chimeras (*n*=4 per group from four individual experiments). Data were analysed by ANOVA followed by Tukey’s *post hoc* test *P<0.05.

*C. rodentium*-infected animals harbouring IL-23-deficient CX_3_CR1^+^ cells acutely succumb to infection. The above data suggest that IL-23 controls otherwise deleterious IL-12 production by CD103^+^ CD11b^−^ DC. IL-12 is known to promote the polarization of naïve T cells into TH1 cells and induce IFNγ secretion by CD4^+^ and CD8^+^ T cells, as well as NK cells that can cause detrimental immunopathology^33^. To investigate whether the uncontrolled IL-12 production by colonic CD103^+^ CD11b^−^ DC triggers such a cascade in our system, we neutralized IFNγ in *Citrobacter*-infected mice that harbour IL-23-deficient CX_3_CR1^+^ cells using an antibody regimen. Indeed, this treatment efficiently rescued infected DTx-treated (CD11c-DTR/*il23a*^−/−^>WT) and (CX_3_CR1-DTR/*il23a*^−/−^>WT) chimeras from pathogen-induced mortality (Fig. 5a,b). Moreover, also the neutralization of IL12p40, that is, the cytokine which is likely involved in the induction of IFNγ^33^, improved the survival of the respective *Citrobacter*-infected animals (Fig. 5a,b).

The kinetics of the observed fatality suggested ILC or a pre-existing T-cell population as IFNγ—source. Fate-mapping studies recently established that TH17 cells can differentiate into pathogenic TH1 cells, both in the brain and gut^34^,^35^,^36^. To evaluate the involvement of this TH17/TH1 cell axis in the *Citrobacter*-infected mice lacking either IL-23 or IL-12/-23, we analysed colonic tissue of whole and mixed BM chimeras generated with *il23a*^−/−^, *il12b*^−/−^, CD11c-DTR and WT BM cells. FACS analysis of T cells isolated from the colon of the various mice, as well as reverse transcription PCR analysis for IL-17A and IFNγ expression indicated a shift towards TH1 cells in IL-23-deficient BM chimeras ((*il23a*^−/−^>WT); Fig. 5c). Similar results were obtained when the IL-23 deficiency was restricted to CD103^−^ CD11b^+^DC and macrophages (DTx-treated (*il23a*^−/−^/CD11c-DTR>WT) chimeras; Fig. 5d). To probe if the observed accumulating TH1 cells derive from TH17 cells, we analysed their CD121a (IL1R1) expression, which has been reported to identify ‘exTH17 cells’ (ref. 35). As shown in Fig. 5e, CD121a was prominently displayed on a fraction of IFNγ^+^ TH1 cells isolated from mice lacking CX_3_CR1^+^ cell-derived IL-23. Of note, IFNγ transcript levels and TH1 frequencies were also elevated in WT chimeras; however, these cells did not display CD121a expression and were resistant to the infection. Collectively, these data suggest that TH17-derived TH1 cells, rather than conventional TH1 cells, are the source of the deleterious IFNγ that causes the severe immunopathology in the absence of the IL-23-mediated mononuclear phagocyte crosstalk.

## Discussion

The aim of this study was to define the differential contributions of the intestinal DC and macrophages in the immediate early innate host defense against *C. rodentium* challenge. Specifically, we identified colonic CX_3_CR1^+^ mononuclear phagocytes, including CD103^−^ CD11b^+^ DC and macrophages, as early producers of the critical IL-23 required for the induction of IL-22 and AMPs^20^. Absence of IL-23, however, not only impaired the innate response, but also revealed the existence of a critical crosstalk between colonic CX_3_CR1^+^ cells and CD103^+^ CD11b^−^ DC. Specifically, we found that IL-23 suppressed IL-12 production by CD103^+^ CD11b^−^ DC thereby preventing fatal immunopathology.

The host response to the A/E mouse pathogen *C. rodentium* can be segregated into early innate reactions and late adaptive immunity^37^. The former depend critically on IL-23, that is required to induce IL-22 production by ILC3 (refs 20, 38). Notch2-dependent small intestinal CD11b^+^ CD103^+^ cells, the likely equivalent of colonic CD103^−^ CD11b^+^ DC, were shown to produce IL-23 upon *Citrobacter* infection^30^. Moreover, these IRF4-dependent cells were reported as the IL-23 source that maintains the steady-state TH17 compartment in this tissue^6^,^7^,^8^. Recent studies further established intestinal macrophages as the IL-23 source^31^. We corroborate these findings by showing that mice that harbour an IL-23 deficiency in colonic CX_3_CR1^+^ mononuclear phagocytes, comprising CD103^−^ CD11b^+^ DC and macrophages, fail to induce IL-22 and AMPs.

Surprisingly, *Citrobacter*-infected animals bearing only an IL-23 (p19) deficiency in all the DCs and macrophages died from the bacterial challenge, whereas animals harbouring a combined IL-12/23 (p40) deficiency were resistant, although both the mice display impaired IL-22 induction. Furthermore, animals with an IL-12/23 mutation restricted to CX_3_CR1^+^ cells succumbed to the infection. Collectively, these data suggested a critical deleterious role of CD103^+^ CD11b^−^ DC-derived IL-12 and ensuing immunopathology as cause of the acute death of the animals. Indeed, antibody-mediated neutralization of IL-12 or IFNγ rescued *Citrobacter*-challenged mice harbouring the IL-23 deficiency.

Aside from orchestrating the early innate host response to *Citrobacter* infection, that is, IL-22 and AMP induction, IL-23 is thus also critically involved in a crosstalk of CX_3_CR1 cells and neighbouring CD103^+^ CD11b^−^ DC. Our conclusion is supported by the fact that (1) CD103^+^ CD11b^−^ DC express IL-23 receptor and seem within the colonic DC compartment to be uniquely sensitive to IL-23; (2) in absence of IL-23, IL12p35 and IL12p40 mRNA expression is significantly increased in CD103^+^ CD11b^−^ DC of *Citrobacter*-challenged mice, as was IL-12 secretion by colon explants. Most importantly, (3) IL-23 receptor-deficient CD103^+^ CD11b^−^ DC displayed prominent IL12p35 mRNA expression over WT DC in the same *Citrobacter*-challenged mice. The fact that IL-23 can interfere with the IL-12 axis has been previously reported in refs 39, 40. Sieve *et al*.^40^ showed that IL-23 could inhibit the IL-12-induced effector function of IFNγ induction by T cells. However the authors argued that this effect was due to a direct antagonistic IL-23 effect on the IL-12 receptor on T cells, rather than an IL23R-mediated effect on IL-12 production by myeloid cells, as shown in this study. Becker *et al*.^39^ showed that in an *in vitro* system IL-23 specifically downregulated IL-12 secretion in LPS-exposed BM culture-derived DC. Focusing on IL23p19-deficient DC, these authors concluded that they observed an autocrine regulatory loop, which they considered responsible for the aggravated colitis observed in IL23p19^−/−^ mice^39^. Our present data support the notion of such a less appreciated, but clinically probably highly relevant immunoregulatory role of IL-23. Moreover importantly, we extend the earlier reports by revealing a novel paracrine regulatory circuit between the specific intestinal mononuclear phagocyte subsets. Of note, this immunoregulatory role is distinct from reported effects of IL-23 on IL-12 receptor signalling in T cells, which limits their IFNγ production^40^. The molecular details underlying the suppressive effect of IL-23 on CD103^+^ CD11b^−^ DC might be related to activation of signal transducer and activator of transcription 3 (STAT3) and thus similar to IL-10 suppression of IL-12 production^41^. However, the exact mechanism remains to be defined.

IL-12 is known to polarize naïve T cells towards a TH1 fate^42^. However, given the short time frame of our experiments, the immunopathology that we observed was unlikely to result from exacerbated adaptive immunity. TH17 cells have been shown to retain plasticity and can under certain pathological conditions, such as experimental autoimmune encephalomyelitis^34^,^35^, but also following *Helicobacter* challenge in the intestine^36^ convert into TH1 cells. Given the presence of TH17 cells in the steady-state colon, we considered these rapidly generated ‘exTH17’ TH1 cells an attractive explanation for the IFNγ pathology we observe. Indeed, we found TH1 cells in our setting of immunopathology to express CD121a (IL1R1) indicative of their TH17 past^35^. However, we cannot include the involvement of other IL-12 responsive IFNγ producers, such as ILC-1 (ref. 43), or additional direct IL-23 effects on ILC (ref. 44).

Intestinal homeostasis and gut resistance to pathogen challenge rely on a balance of pro- and anti-inflammatory processes. This ‘homeostastic inflammation’ is established in response to the constant microbial product exposure of this unique organ^45^. Our findings highlight the interplay of these processes by corroborating the role of myeloid cell-derived IL-23 as an inducer of IL-22, which is known to promote mucosal healing and stabilize barrier integrity. Importantly though, our data indicate that IL-23 curbs the simultaneously potential damaging immunopathology caused by IL-12 production. Of note, our data do not argue against the importance of IL-22 in the protection against *Citrobacter* challenge, which is well documented^18^,^20^. However, we show that under certain conditions, such as the absence of IL-12 production by CD103^+^ CD11b^−^DC, *Citrobacter*-challenged mice can handle an impaired IL-22 response, probably since epithelial injury is less severe.

Genome-wide association studies have revealed a link between allelic variants of the IL-23 receptor, its signalling and the risk to develop Crohn’s disease or ulcerative colitis, although the underlying mechanistic details remain to be elucidated^46^,^47^. Given the appreciated role of IL-23 in the control of ILC3 and TH17 biology, efforts to understand the reason for genetic IBD predispositions are currently largely focused on the effect of this cytokine on innate and adaptive lymphocytes. Though not mutually exclusive, our results suggest that the IL-23 receptor variants could also affect a critical regulatory circuit between myeloid cells that—when disrupted—might result in deleterious hyperactivity of intestinal DC. Our results highlight the intricacy of the regulatory circuits that exist in the intestinal lamina propria to maintain immune protection and prevent collateral damage. Moreover, these findings should be of clinical importance highlighting a previously under-appreciated immunoregulatory role of IL-23 on mononuclear phagocytes.

## Methods

### Mice

This study involved the use of the following mice, all on C57BL/6 background: B6.129S1-*Il12b*^*tm/Jm*^/J mice (*p40*^−/−^)^48^ and B6.IL23p19^tm^ (*p19*^−/−^) mice^49^; CX_3_CR1^Cre^mice^27^ (deposited at the European Mouse Mutant Archive,EM:06349; JAX Stock No. 25524 B6.C-Cx3cr1<tm1.1(cre)Jung>/J); Rosa26-DTR (iDTR)^28^; CD11c-DTR mice^24^;IL-23R^gfp^ mice^32^. To analyse recombinase activity of *Cx3cr1*^*cre*^ mice the animals were crossed with *Rosa-26-rfp* (ref. 50).For BM chimera generation, C57BL/6 mice were lethally irradiated with 950 rad. The next day, 5 × 10^6^ BM cells isolated from the femora and tibiae of donor mice were intravenously injected into the irradiated recipient mice, which were kept at rest for 6–8 weeks before the experiment. Animals were maintained under specific pathogen-free conditions and handled according to the protocols approved by the Weizmann Institute Animal Care Committee as per international guidelines.

### *Citrobacter* infection and assessment of bacterial load

Mice were gavaged with a spontaneous naladixic acid-resistant derivative of the strain *C. rodentium*ICC168 (*Citrobacter freundii* biotype 4280), that is kanamycin-resistant and bioluminescent, the strain ICC180 (ref. 23), a kind gift of Gad Fraenkel (Centre for Molecular Bacteriology and Infection, Division of Cell and Molecular Biology, Imperial College London, London, United Kingdom). Mice were gavaged with 0.2 ml of PBS containing 10^9^ c.f.u. Bacteria were grown at 37 °C in Luria Bertani broth or on Luria Bertani agar with kanamycin (50 μg ml^−1^) After 3 h the bacterial density was assessed by measuring its absorbance at an optical density of 600 nm and confirmed by plating of serial dilutions. For the assessment of bacterial load, mice were anaesthetized and imaged using IVIS (Xenogen, Almeda, CA). Greyscale reference images taken under low illumination were collected and overlaid with images capturing the emission of photons from the bioluminescent *C. rodentium* using LIVING IMAGE software (Xenogen). For the analysis of the colon, mice were euthanized and imaged as above.

### Cell preparations

For isolation of lamina propria DC and macrophages, intestinal epithelial cells were removed by incubation with the HBSS containing DTT and 5% of fetal calf serum and shaking in a 37 °C incubator (300 r.p.m.) for 40 min. Intestinal pieces were digested in 5 ml HBSS containing 10% FBS, 1.2 mg ml^−1^ of collagenase VIII (Sigma) and shook (300 r.p.m.) at 37 °C for 40 min. Digested colon tissue was passed through a 80-μm mesh and cells were collected by centrifugation at 1,400 r.p.m. for 10 min at 4 °C. For isolation of intestinal T cells, lamina propria cells were after the Collagenase digest fractionated on a Percoll density gradient (40 and 80%). To allow for the intracellular cytokine staining of TH17 cells and TH1 cells, IL-17A and IFNγ production was triggered and accumulated by PMA and ionomycin (Sigma) treatment for 2 h and treatment with Brefeldin A and Monensin (eBioscience) for 2 h at 37 °C 5% CO_2_. Cells were permeabilized and fixed for the intracellular staining by the BD perm/fixation kit.

### Antibody-mediated cytokine neutralization

For the neutralization experiments, indicated mice were intraperitoneally injected with anti-IFNγ-specific mAb (Clone, XMG1.2) or anti-IL12p40-specific mAb (C17.8) on day 0, 2, 4, 6 and 8 after *C. rodentium* infection at a dose of 1 mg per mouse. The control groups received isotype control IgG2a mAb.

### Cell ablation

For cell depletion in (CX_3_CR1-DTR>WT) BM chimeras, 25 ng g^−1^ body weight diphtheria toxin (DTx; Sigma-Aldrich) was injected IP every 48 h for 5 days and the mice were euthanized 18–20 h after DTx injection. For prolonged cell ablation in the functional assays, the dose was reduced to 12.5 ng g^−1^ body weight after the first 5 days (day −1 was one day before infection). (CD11c-DTR>WT) BM chimeras were injected 12.5 ng g^−1^ in the first 5 days and 6.25 ng g^−1^ body weight until the end of the assay.

### cDNA synthesis and real-time PCR

Total RNA was extracted from the colon using the Gentle-MACS (MiltenyiBiotec) and RNA tissue kit (5prime) and from the sorted cells with the RNeasy Micro Kit (Qiagen). cDNA synthesis was performed with 0.5—2 μg of total RNA with High Capacity RNA-to-cDNA Kit (Applied Biosystems). Real-time PCR was performed with SYBR Green master mix (Applied Biosystems). Expression of each target gene was normalized to that of TATA binding protein using the comparative (ΔΔCT) method.

### Histology

Tissues were fixed in 4% paraformaldehyde overnight at 4 °C, embedded in paraffin, sectioned and stained with periodic acid-Schiff. Slides were evaluated using an Olympus BX51 microscope, and image acquisition was conducted with the Olympus DP70 camera and DP-Manager software. The percent area of goblet cells and colonic hyperplasia were calculated using a technique established previously^51^.

### Flow cytometric analysis

The following fluorochrome-labelled monoclonal antibodies and staining reagents were used according to manufacturer’s protocols: lamina propria cells were stained with anti-CD11c-APC, anti-CD64-APC (eBioscience), anti-CD11b-PerCP Cy5.5 (Biolegend), anti-CD103-PE/Horizon 421(BD Pharmingen), anti-MHC II-PE-CY7, anti-CD24- PE-CY7 (eBioscience), anti-CD45.2-FITC (eBioscience), anti-CD45.1-Pacific blue (Biolegend) and 4′,6-diamidino-2-phenylindole. TH17 and TH1 cells were stained with anti-IL-17A-PE, anti-CD3-biotin, SA-PerCP, anti-CD4-APC, anti-CD8-FITC, anti-CD45-Pacific Blue anti-IFNγ-PE-CY7 and anti-CD121A-PE. The cells were analysed with LSR Fortessa flow cytometer (BD) or sorted with a FACSAria machine (BD). Flow cytometry analysis was done with the FlowJo software.

### Analysis of colon explant cultures and ELISA

The colons of mice were flushed with RPMI and open along a longitudinal axis. Thereafter, 3-mm^2^ punch biopsies were obtained from the distal colon and incubated for 24 h in RPMI supplemented with 10% fetal calf serum and antibiotics (one punch biopsy per 100 ml medium). Supernatants were collected and kept frozen until assessed. Quantitative evaluation of IL-12 and IFNγ levels in supernatant were done using an ELISA modification, with spectrally distinguishable beads (Spherotech) as solid phase. Primary antibodies were covalently linked to the beads, with spectrally different beads linked to antibodies against a unique analyte. The various coated beads were incubated simultaneously with the supernatant and secondary biotinylated antibodies for 2 h, washed and stained with streptavidin-PE. Bead fluorescence level was used to separate the different analytes and then compared with a standard curve for quantification. The beads were analysed with LSRFortessa flow cytometer (BD).

### Statistical analysis

Data were analysed by analysis of variance followed by Tukey’s *post hoc*, two-tailed *t*-test, and long-rank analysis on the Kaplan–Meier plots using GraphPad Prism 4 (San Diego, CA). Data are presented as mean±s.e.m. values of *P*<0.05 were considered statistically significant. *P<0.05, **P<0.01, ***P<0.001.

## Author contributions

T.A. conducted the experiments; N.Y., A.W., L.B. and D.J.C. provided critical reagents; A.M. S. R.-Z. and N.L. helped with experiments; S.Y. And K.-W.K generated mice; T.A. and S.J. prepared the manuscript; S.J. directed the research.

## Additional information

**How to cite this article**: Aychek, T. *et al*. IL-23-mediated mononuclear phagocyte crosstalk protects mice from *Citrobacter rodentium*-induced colon immunopathology. *Nat. Commun*. 6:6525 doi: 10.1038/ncomms7525 (2015).

## Supplementary Material

Supplementary Information Supplementary Figures 1-6 and Supplementary Table 1
