# Supplementary material for: IL-23-mediated mononuclear phagocyte crosstalk protects mice from Citrobacter rodentium-induced colon immunopathology
Source: Nat Commun. 2015 Mar 12;6:6525. doi: 10.1038/ncomms7525 (PMC4382688; doi:10.1038/ncomms7525)
Supplement: Supplementary Information — Supplementary Figures 1-6 and Supplementary Table 1 [file ncomms7525-s1.pdf]

Supplementary Information

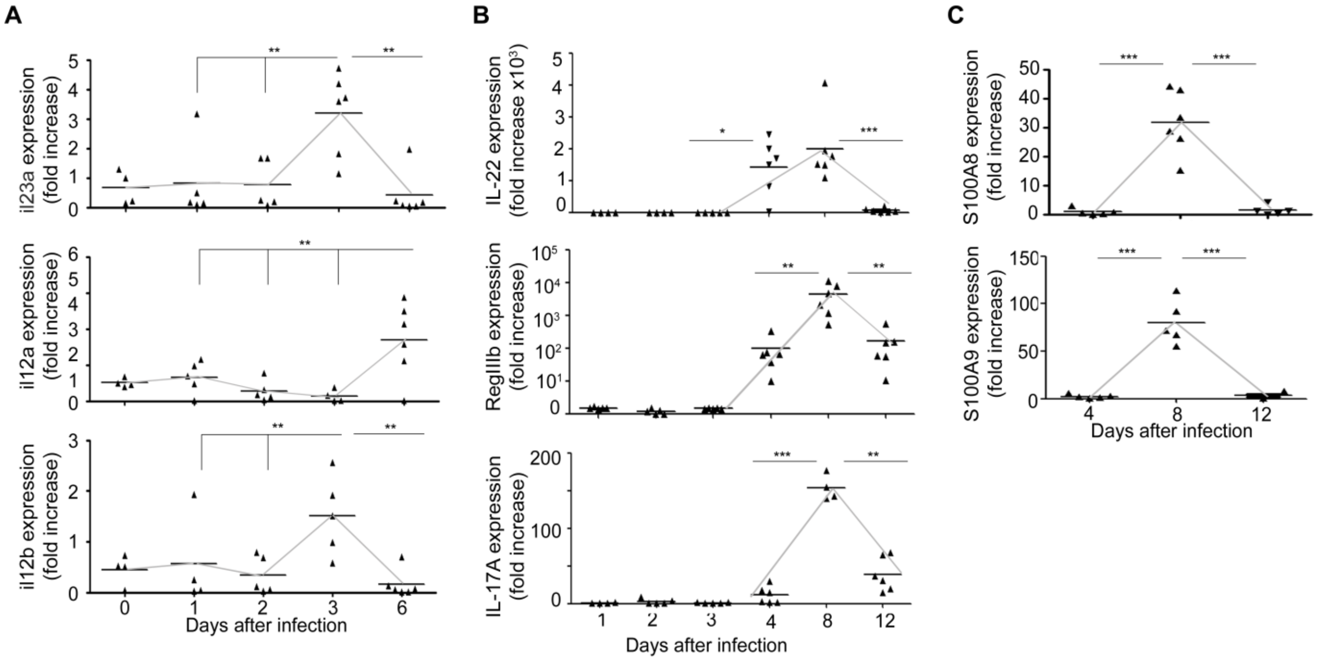

Supplementary Figure 1. Cytokine dynamics during the early host response to *C. rodentium* infection in WT mice.

Mice were infected with10^9 CFU of *C. rodentium* by gavage and a time-course RT-PCR analysis of infected colonic tissue was performed. (A) *il23a* and *il12b* were upregulated on day 3, *il12a* was upregulated on day 6. (B) IL-22 was found upregulated on day 4 and day 8, waning by day 12. IL-17A, RegIIIβ, S100A9 and S100A8 (C) were found upregulated on day 8 (n=4-6 per group from 3 individual experiments). The data are represented as fold increase normalized to non-starved and non-infected mice. Asterisk P<0.05, two asterisks P<0.01, three asterisks P<0.001.

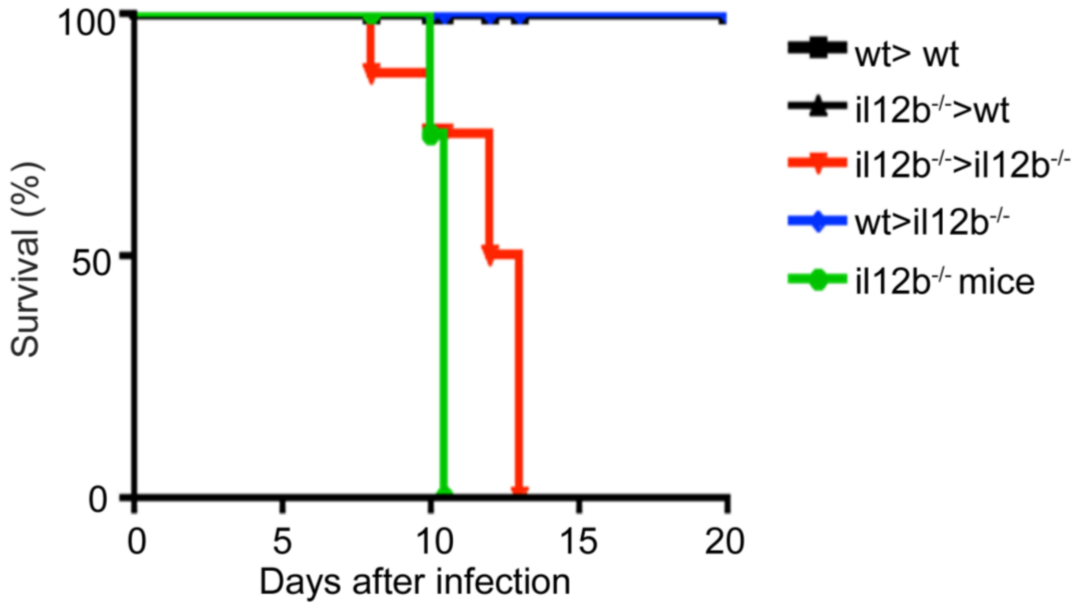

Supplementary Figure 2

Survival curves of *Citrobacter*-challenged [WT > WT], [*il12b* $^{-/-}$> WT], [*il12b* $^{-/-}$>*il12b* $^{-/-}$] and [WT > WT] BM chimeras, as well as *il12b* $^{-/-}$ mice. (n = 6 per group).

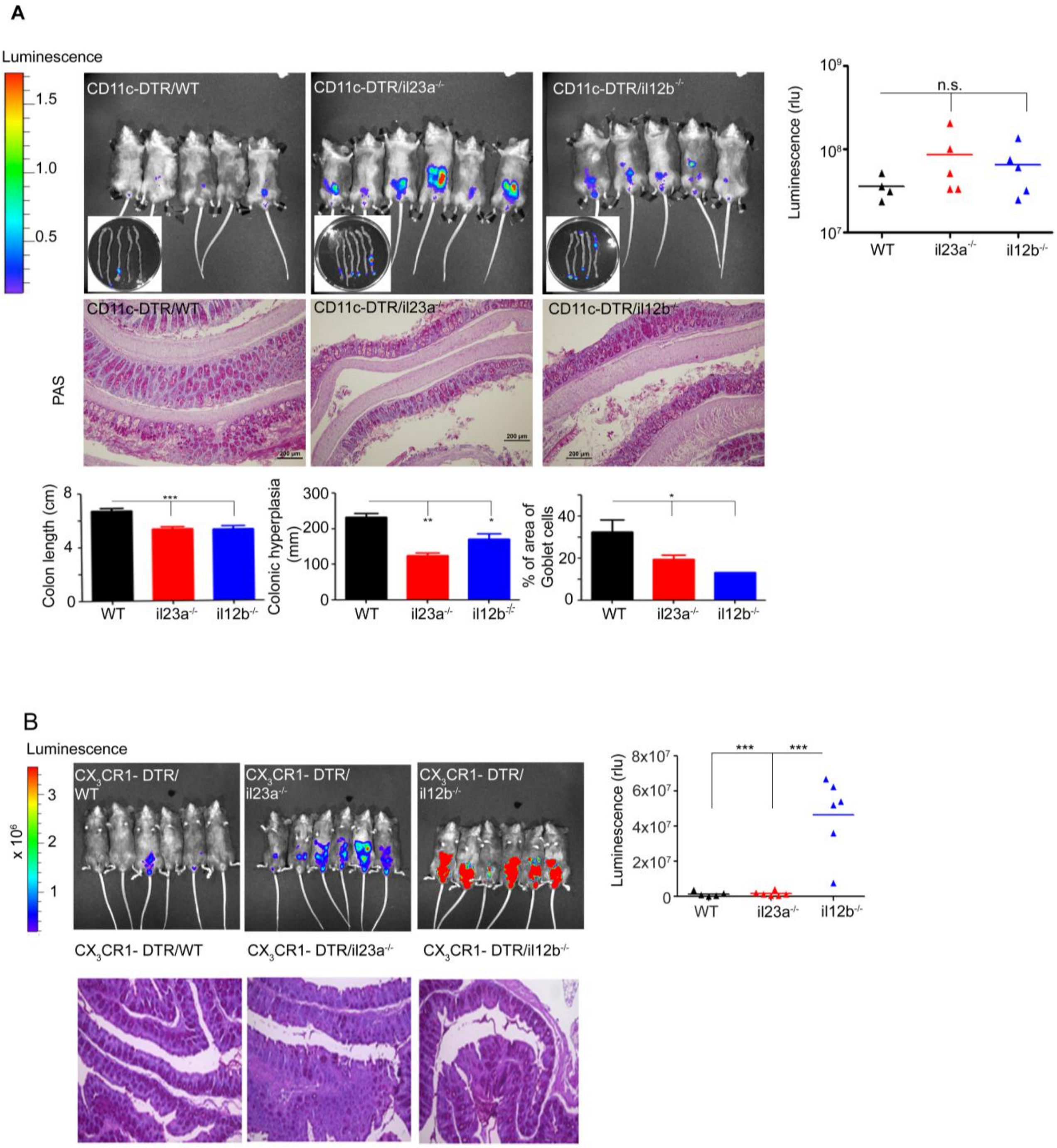

Supplementary Figure 3

(A) Analysis of bacterial load and goblet cell status (PAS staining) at day 7; note loss of enterocytes and decrease in mucosal hyperplasia in [*il23a*$^{-/-}$/CD11c-DTR > WT], [*il12b*$^{-/-}$/CD11c-DTR > WT] and [WT / CD11c-DTR >WT] BM chimeras. Bar diagrams indicate colon lengths, colonic hyperplasia and goblet cell abundance.

(B) Bacterial load and histology of *C. rodentium* - infected DTx-treated mixed [*il23a*$^{-/-}$/ CX_3CR1-DTR > WT], [*il12b*$^{-/-}$/ CX_3CR1-DTR > WT] and [WT / CX_3CR1-DTR > WT] BM chimeras on day 8 post infection (n=5-6 per group from 3 individual experiments).

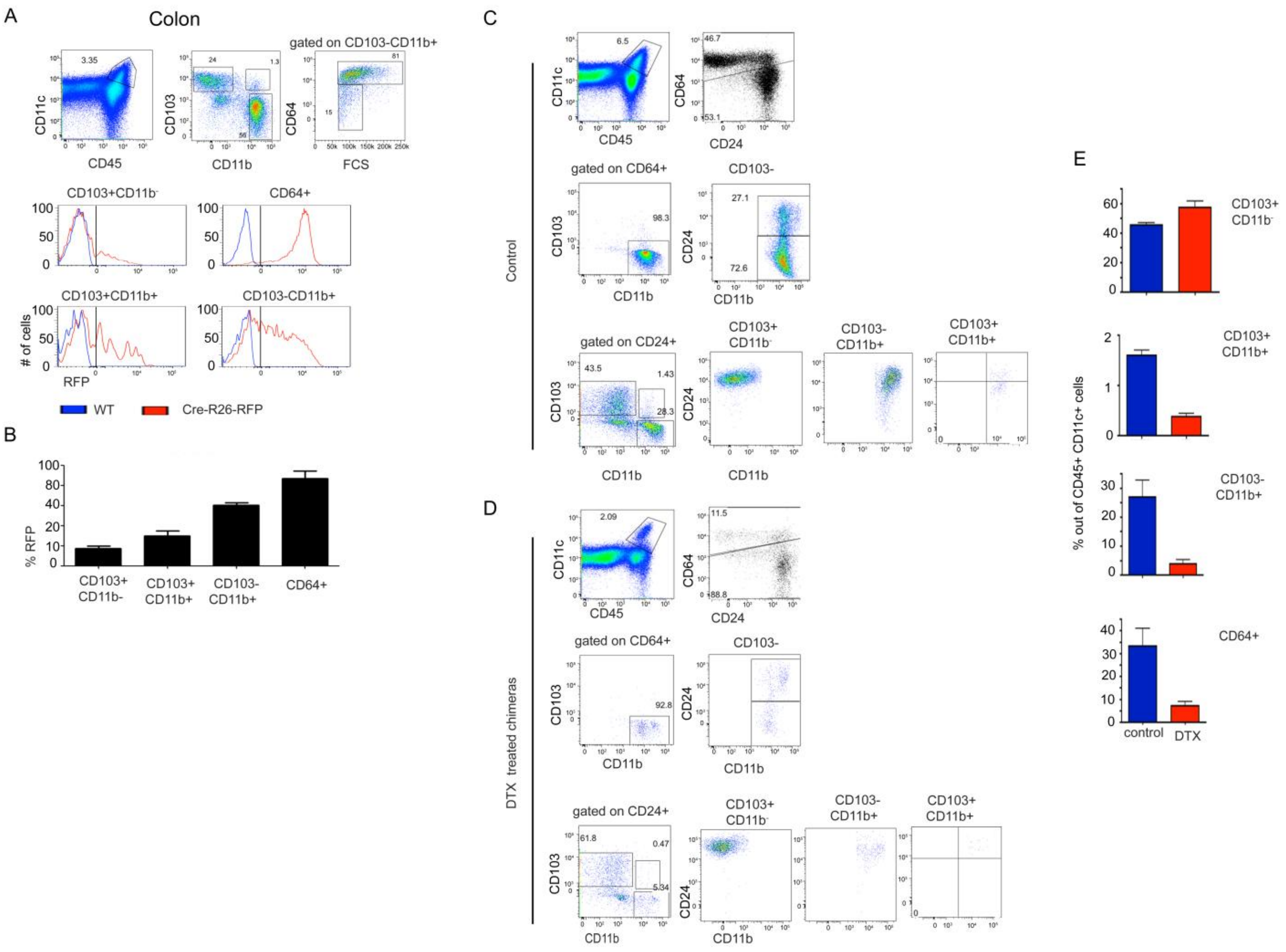

Supplementary Figure 4

(A) Flow cytometric analysis of colonic mononuclear phagocyte compartment of $CX_3CR1^{Cre/+}$:R26-RFP mice for reporter gene expression.

(B) Bar diagram summarizes data (n=3 per group from three individual experiments).

(C, D) Flow cytometric analysis of colonic mononuclear phagocyte compartment of DTx-treated [CX_3CR1-DTR > WT] BM chimeras 5 days after DTX treatment.

(E) Bar diagram summarizes data (n=3 per group from three individual experiments).

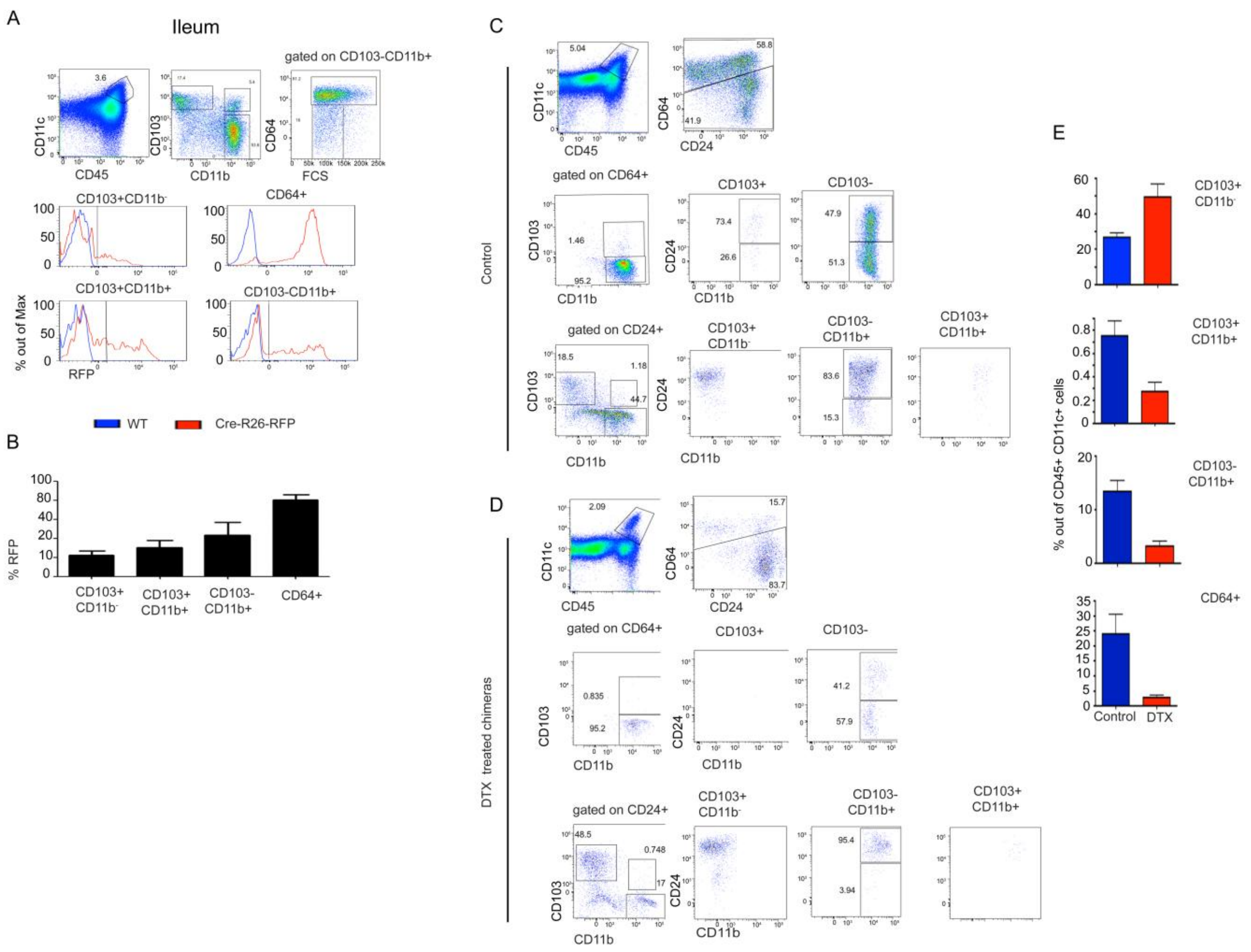

Supplementary Figure 5

(A) Flow cytometric analysis of ileal mononuclear phagocyte compartment of $CX_3CR1^{Cre/+}$:R26-RFP mice for reporter gene expression.

(B) Bar diagram summarizes data (n=3 per group from three individual experiments).

(C, D) Flow cytometric analysis of ileal mononuclear phagocyte compartment of DTx-treated [CX_3CR1-DTR > WT] BM chimeras.

(E) Bar diagram summarizes data (n=3 per group from three individual experiments).

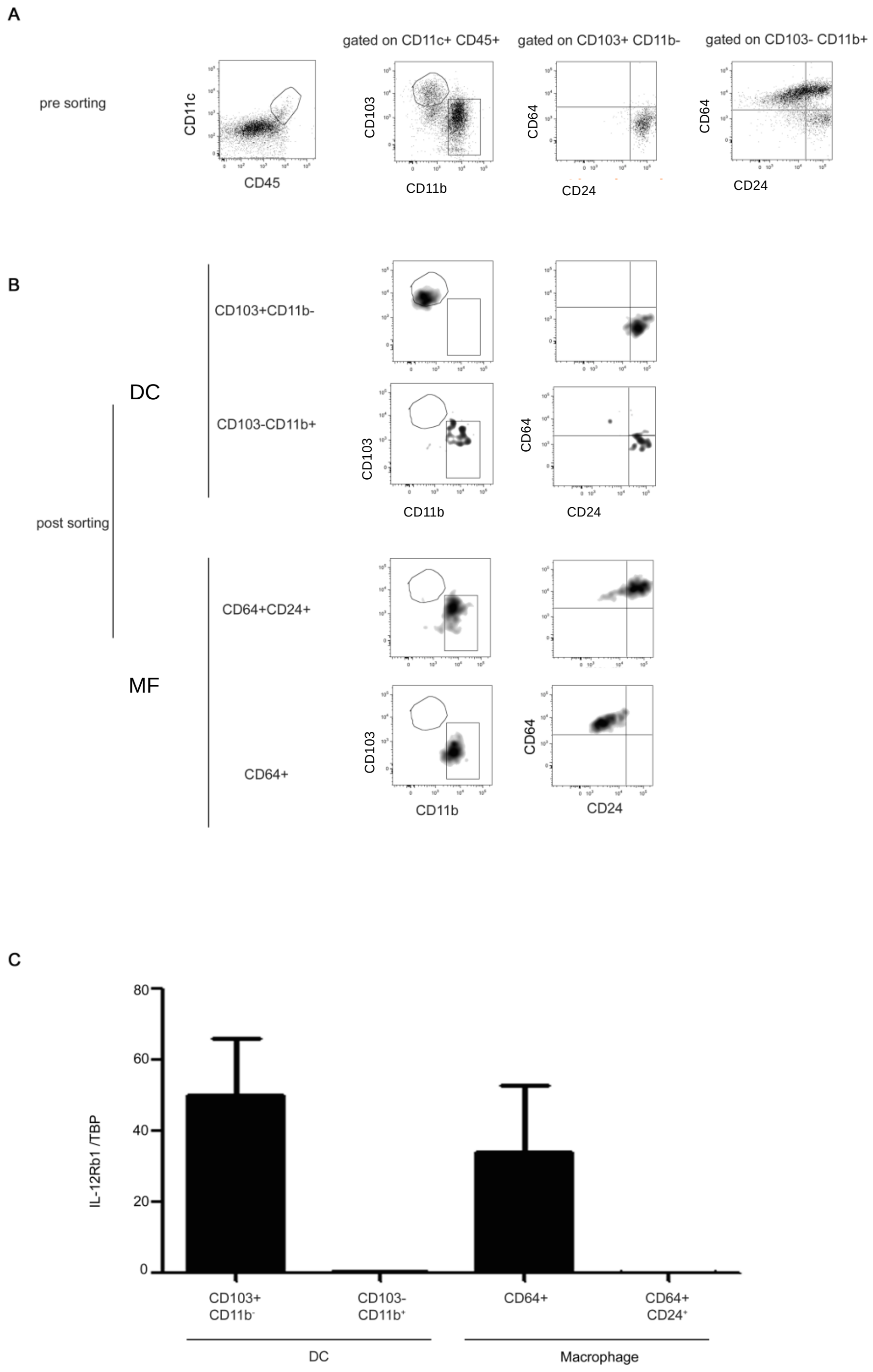

Supplementary Figure 6

Flow cytometry sorting strategy for colonic lamina propria resident DC and macrophages. Representative flow cytometry images used to identify and sort colonic DC and macrophages of WT mouse. **(A)** presort analysis; **(B)** postsort analysis

(C) RT-PCR analysis of *IL12rb1* expression on FACS sorted lamina propria DC and macrophages isolated from infected WT mice three days post infection. Data are representative of 3 independent experiments with DC and macrophages pooled from 7-10 mice.

Supplementary Table 1

Real-time PCR primers

The sequences for primers used in this study are as follows:

Primers	Forward	Reverse
**IL-17A**	5'-GCT CCA GAA GGC CCTCAG A-3'	5'-CTT TCC CTC CGC ATT GAC A-3'
**IL-6**	5'-TCC AAT GCT CTC CTA ACA GAT AAG-3'	5'-CAA GAT GAA TTG GAT GGT CTT G-3'
**IL-12p40**	5'-ACA TCT ACC GAAGTC CAA TGC A-3'	5'-GGA ATT GTA ATA GCG ATC CTG AGC-3'
**IL-23p19**	5'-GGT GGC TCA GGG AAA TGT-3'	5'-GAC AGA GCA GGCAGG TAC AG-3'
**IL-12 p35**	5'- GCC ACC CTT GCC CTC CTA A -3'	5'- GGT TTG GTC CCG TGT GAT GTC -3'
**IFNγ**	5'- ATG GCT GTT TCT GGC TGT TAC TG -3'	5'- ATC TGG CTC TGC AGG ATT TTC A -3'
**S100A8**	5'- TGT CCT CAG TTT GTG CAG AAT ATA AA-3'	5'-TCA CCA TCG CAA GGA ACT CC-3'
**RegIIIβ**	5'- ATG GCT CCT ACT GCT ATG CC-3'	5'- GTG TCC TCC AGG CCT CTT T-3'
**IL-22**	5'-TCC GAG GAG TCA GTG CTA AA-3'	5'-AGA ACG TCT TCC AGG GTG AA-3
**TBP**	5'-GAAGCTGCGGTACAATTCCAG-3'	5'-CCCCTTGTACCCTTCACCAAT-3'
**IL-23R**	5'-TGGTGTCACGGAGGAATCACAAGT3'	5'-AGATTCCTTGGTCGGCAGTGCTTA3'
**S100A9**	5'-GGT GGA AGC ACA GTT GGC A-3'	5'-GTG TCC AGG TCC TCC ATG ATG-3'
**IL-12Rb1**	5'-CCCCAGCGCTTTAGCTTT-3'	5'GCCAATGTATCCGAGACTGC-3'
